# Metal–Flavonoid Interactions—From Simple Complexes to Advanced Systems

**DOI:** 10.3390/molecules29112573

**Published:** 2024-05-30

**Authors:** Paulina Katarzyna Walencik, Renata Choińska, Ewelina Gołębiewska, Monika Kalinowska

**Affiliations:** 1 Institute of Agricultural and Food Biotechnology-State Research Institute, Rakowiecka 36, 02-532 Warsaw, Poland; renata.choinska@ibprs.pl; 2 Department of Chemistry, Biology and Biotechnology, Faculty of Civil and Environmental Sciences, Bialystok University of Technology, Wiejska 45E Street, 15-351 Bialystok, Poland; e.golebiewska@pb.edu.pl

**Keywords:** hybrid materials, redox, flavonoids, transition metals

## Abstract

For many years, metal–flavonoid complexes have been widely studied as a part of drug discovery programs, but in the last decade their importance in materials science has increased significantly. A deeper understanding of the role of metal ions and flavonoids in constructing simple complexes and more advanced hybrid networks will facilitate the assembly of materials with tailored architecture and functionality. In this Review, we highlight the most essential data on metal–flavonoid systems, presenting a promising alternative in the design of hybrid inorganic–organic materials. We focus mainly on systems containing Cu^II/I^ and Fe^III/II^ ions, which are necessary in natural and industrial catalysis. We discuss two kinds of interactions that typically ensure the formation of metal–flavonoid systems, namely coordination and redox reactions. Our intention is to cover the fundamentals of metal–flavonoid systems to show how this knowledge has been already transferred from small molecules to complex materials.

## 1. Introduction

The role of metal ions in natural systems has been studied extensively for years, discovering step by step their importance in enzyme catalysis, signal transduction, electron transfer, and synaptic transmission. The value of metal ions for cellular homeostasis is indisputable [1,2]. Apart from being cofactors, metal ions have been found to be valuable in the construction of advanced supramolecular systems, both natural and manufactured. When producing artificial systems using metal ions, three types of interactions are postulated: cation-π, coordination, and redox interactions. A pronounced feature of artificial hybrids is their ability to manipulate their synthesis and linking since their structure, folding, and mechanical properties are directed by the type of metal ion used and its coordination. This provides an advantage of hybrid materials over standard organic polymers, especially since strategies for obtaining sequence-defined organic macromolecules are still scarce and polymerization tends to yield rather polydisperse systems.

**Figure 1:**
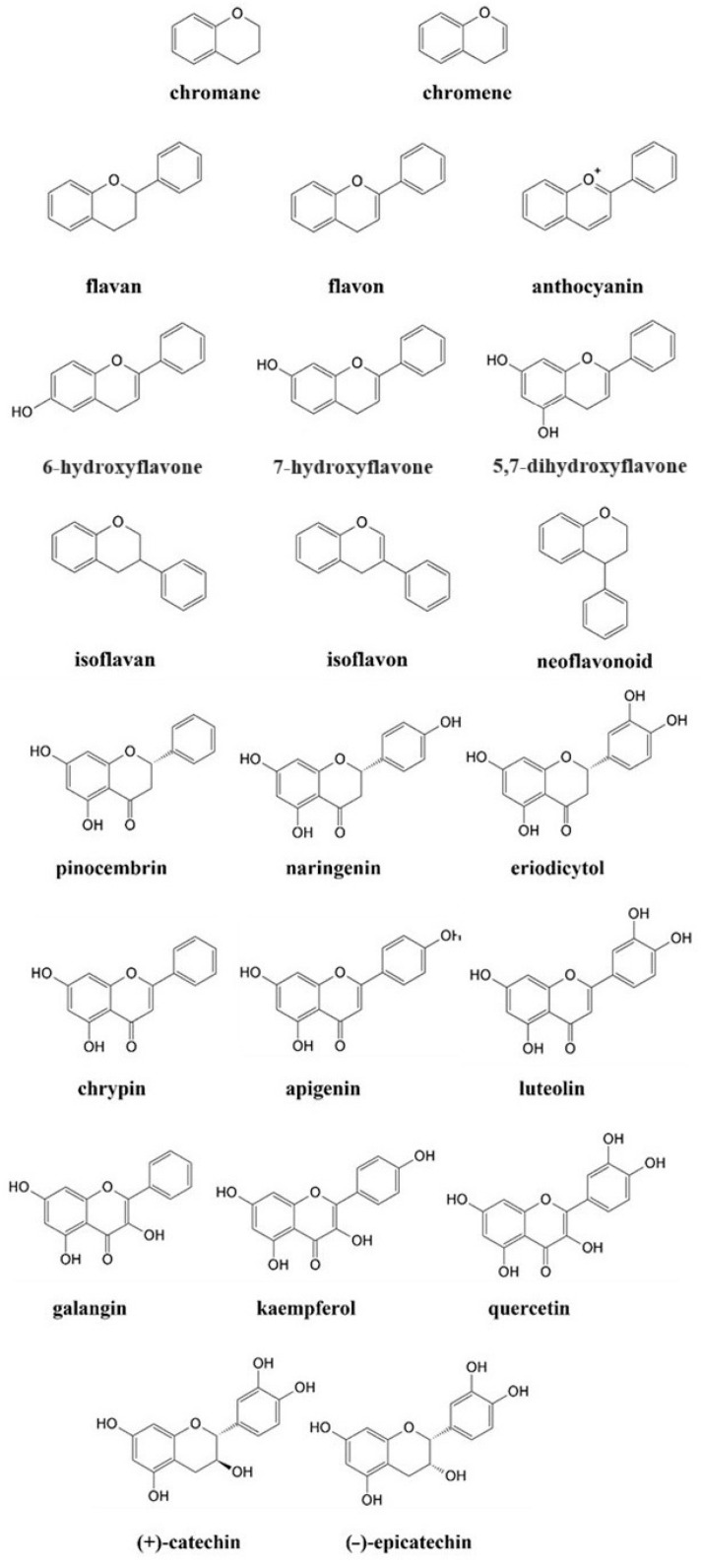
Chemical structure of basic compounds and selected flavonoids.

Materials consisting of natural phenolic ligands have attracted great attention owing to their abundance in plants, their efficiency in coordination, and their high reactivity during the fabrication of metal–organic networks [3,4]. Plant phenolic compounds (Figure 1) are mostly used in nutrition and pharmacy due to their antioxidant, anti-inflammatory, antibacterial, and anticancer activities [5,6]. Independently, more and more attention has been placed on the role of flavonoids (FLs) as building components [7,8,9]. 

FLs, one of the most important groups of plant phenolic compounds, fulfill most of the criteria for chemicals used in the engineering of supramolecules, i.e., abundance, processability, and high biocompatibility. Their structural diversity enables the construction of more sophisticated and tailored systems. The self-assembly of flavonoid-based materials is usually directed by transition metal ions, and in this case, catalysis proceeds involving coordination or redox reactions. The final mechanism depends on the structure of FLs and the nature of metal ions, as well as environmental conditions like solvent type, temperature, or pH [10,11]. Additionally, there is a decent chance that alterations in the environment would convert typical non-covalent interactions into redox conversions. To this end, knowledge of the fundaments under which FLs are active is required, due to the uniqueness of each flavonoid and its specific electron distribution, reactivity, and redox properties. These aspects have been discussed in detail in the first section of the Review.

Electron-rich FLs interact with a wide range of transition metal ions, but the results and character of such interactions are directed by many elements. Comprehensive reviews are available in this area, but they are largely focused on complexes exhibiting in vitro effects [12,13]. There is a current need to unravel the principles and physicochemical properties of more advanced metal–flavonoid systems, specifically including the most common interactions like coordination and redox. Another question is to distinguish the conditions under which these interactions proceed, namely when metal ions guide the donor–acceptor transfer of electrons and when they promote reduction–oxidation effects. This Review collects major findings on the interactions of diverse FLs with two transition metal ions, Cu^II^ and Fe^III^. Cu^II^ and Fe^III^ have been widely exploited to generate hybrid metal–flavonoid compounds [8,14,15]. An understanding and mastering of the principles that rule simple metal–flavonoid complexes are crucial for the engineering of more advanced materials, and this issue is covered in the next sections of this Review.

Metal–flavonoid networks are usually formed using coordination bonds. Alternatively, cross-linking can be initiated by redox reactions, which later are followed by typical coordination [4,16,17]. Therefore, precision in the fabrication of hybrid materials requires careful selection of both organic and inorganic segments. Even small alterations in micro-organization may influence the macroscale by changing the stiffness, durability, and stability of materials. Beyond the structure and reactivity of reagents, the fabrication of materials is affected by environmental factors like temperature, preparation time, or type of solvent used [16]. All these elements are essential to consider while planning the architecture and functionality of the final product. In this context, the systematization of data is necessary for the designing procedure. An overview of recent findings will also give a fresh perspective on these issues that need further exploration. The self-assembly of metal–flavonoid materials using FLs as building blocks and Fe^III^ and Cu^II^ ions as cross-linking initiators is discussed in the last section of the Review.

Metal-based complexes provide valuable paradigms for the design of artificial hybrid materials, and the use of plant-based ligands supports the development of the green chemistry sector [7,17]. Although the preparation of metal–flavonoid systems has not been fully optimized, recent findings make this idea foreseeable and promising. Thus, this Review summarizes the most important fundamentals of metal–flavonoid interactions along with the latest findings in elaborating hybrid materials to inspire the future design of complex matter. The review material includes the literature from the last two decades. The search was conducted in electronic databases, such as Google Scholar, Scopus, PubMed, MDPI, and Elsevier. The keywords used in our search were ‘redox’, ‘flavonoids’, ‘transition metals’, ‘Cu-flavonoid complexes’, ‘Fe-flavonoid complexes’, ‘hybrid materials’, ‘metal-flavonoid systems’, ‘coordination’, and ‘metal phenolic-networks’. This comprehensive search strategy ensures that our Review is thorough and unbiased, incorporating a wide range of relevant literature.

## 2. Redox Activity of Flavonoids

From the molecular perspective, redox activity is provided primarily by phenolic hydroxyl groups and can be modulated by changing their number and location [18,19]. No less important are other fragments like aromatic rings, C=O groups, or donors and acceptors of hydrogen bonds, which improve the distribution of charge and stability of an oxidized product [20,21]. Redox potential is also affected by the number and location of remaining substituents, like methyl, phenyl, esters, or saccharides [22,23,24].

**Figure 2:**
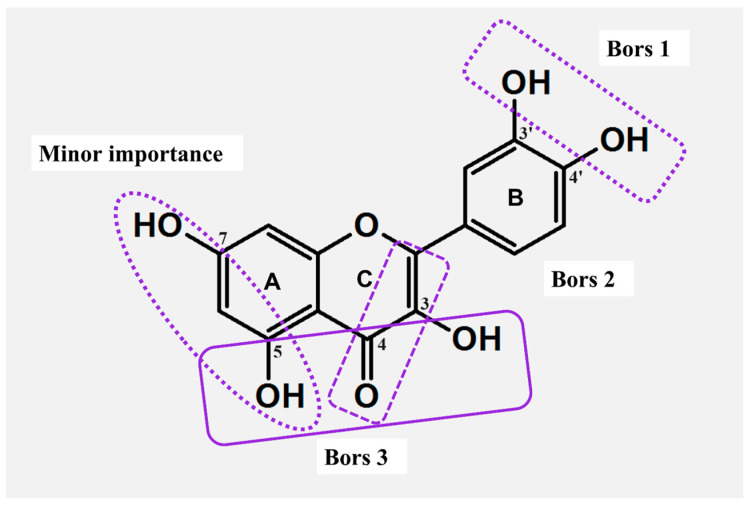
Active sites responsible for the formation of metal–flavonoid systems and structural features of major and minor importance for the antioxidant actions (Bors criteria).

The comparison of pinocembrin, naringenin, and eriodictyol (Figure 1) illustrated how the number of OH substituents attached to the B ring (Figure 2) affects the scavenging activity of the entire compound. In the DPPH (2,2-diphenyl-2-picrylhydazyl) assay, the molecule of eriodictyol with a catechol moiety illustrated an advantage over the remaining flavones. As observed, the ortho arrangement of OH groups is not random but is essential for this higher activity [25,26]. Similar conclusions were drawn by applying another group of flavones:chrysin (no OH in the B ring), apigenin (phenol moiety), and luteolin (catechol moiety) (Figure 1). In this case, the most effective compound against DPPH^•^, FRAP (Ferric Reducing Antioxidant Power Assay), and ORAC (Oxygen Radical Absorbance Capacity Assay) agents was luteolin, the only compound with a catechol fragment [26,27]. The parameters of ΔH_BDE_ (bond dissociation enthalpy) and ΔG≠ (the Gibbs activation energy barrier) calculated using the DFT (Density Functional Theory) method confirmed that the compounds with catechol moiety are the most efficient scavengers [28].

The opposite conclusion was drawn for the hydroxylation of the A ring (Figure 2). For instance, in ABTS (2,2′-azino-bis(3-ethylbenzothiazoline-6-sulfonic acid)) and DPPH assays, the monohydroxylated FLs, like 6-hydroxyflavone, 7-hydroxyflavone, and 5,7-dihydroxyflavone (Figure 1), were able to provide an inhibition of radicals comparable to that of the non-hydroxylated flavone [29]. Furthermore, compounds with meta-OHs attached to the A ring showed moderate antioxidant activity compared to their isomers with the hydroxylated B ring (Figure 2) [20]. A greater scavenging effect was achieved only when the catechol group was incorporated into the A fragment. In ABTS, DPPH, and FRAP tests, such FLs demonstrated almost identical antioxidant properties like compounds bearing the ortho-hydroxylated B-ring (Figure 2) [26].

The overall structure of FLs is not conjugated; instead, the entire molecule is split into two electron-rich fragments, labeled AC and BC. The charges of AC and BC are strictly isolated from each other, and such isolation enables a free flow of electron density, which is essential for the production of stable radicals. Specifically, compounds with the C=O group in the C ring demonstrate a stronger cross-conjugation effect [20]. For example, studies involving galangin and 3,5,7-trihydroxychromone revealed higher activity of the primer compound against the DPPH^•^ radical, precisely because of the presence and charge of the BC complex. In addition, a strong dominance of BC over the AC was achieved in every studied case, and even hydroxylation of the latter structure did not change the results [20,30]. As deduced, the linking in BC via the σ-bond ensures free rotation, thus the B ring does not share the same plane as the chromone or chromane units. Rotation enhances the π-π conjugations in the BC complex, providing different resonance formulas, and improving the stability of a formed radical [20,31].

Higher scavenging properties were also reported for FLs with an unsaturated C ring (Figure 2). In this case, the C2-C3 double bond ensures the delocalization of electrons, thus supporting the reactivity of B and enhancing the stability of its aryloxyl radical [31]. The importance of the unsaturated C was proven in DPPH and ABTS assays by applying naringenin chalcone, apigenin, and naringenin. The stronger activity of the former stemmed from a greater delocalization of charge, which supported the formation of six different resonance formulas. Respectively, naringenin, having a saturated C ring, was capable of forming only four resonance species. As deduced, 2,3-hydrogenation impedes π-π conjugation in the BC complex, thus resulting in a lower number of resonance formulas and limiting the antioxidant potential of individual FLs [32].

Among all the concepts proposed to classify the structure–activity relationship (SAR), the criteria presented by Bors are the most widely used (Figure 2). Two of them outline the impact of the hydroxylation of B (Bors 1) and the 2,3-dehydrogenation of C (Bors 2). The third criterion (Bors 3) associates the interaction among the substituents in the AC complex, namely between the 4-C=O group of C and OHs of A [33]. In this case, the comparison of flavan-3-ol and flavonol illustrates the importance of the 4-C=O in the formation of stable radicals. Another critical aspect is the planarity of molecules. As shown, the 4-keto group of C and its interaction with 3-OH from A introduces planarity in the structure of flavonols, thus improving the aromaticity of the AC complex and delocalization of the electronic charge [31].

Clear differences in activity were observed for compounds bearing a diverse linking in the BC complex. As evidenced, the linking with C3 ensured efficient π-conjugation in the molecule of isoflavonoid, and such bonding yielded better antioxidants. Conversely, weaker activity was observed for FLs that contain linkage through C2. For instance, an isomeric couple of genistein (isoflavonoid) and apigenin (flavonoid) demonstrated diverse activity against ABTS^•+^, with a clear dominance of the first compound reported. Also, a higher reducing potential was reported for isoflavonoids by using standard electrochemical methods [34]. Additionally, the most active isoflavonoids fulfill the same Bors criteria as FLs. In this case, 3′OH and 4′OH from B had the lowest BDEs, thus isoflavonoids with such substituents ensured the most efficient scavenging [35].

**Figure 3:**
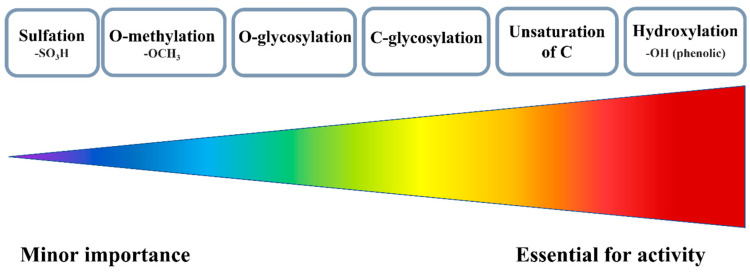
Various substituents and their importance for the antioxidant activity of FLs.

The reactivity of FLs can be modulated by naturally occurring modifications such as -methylation, -glycosylation, -sulfation, and -acylation [18,36]. All of these reactions have been established over the years to help in the adaption of plants to the environment (Figure 3) [37].

There are two possible methylation patterns, i.e., O-methylation, and C-methylation. In general, O-methylation, which mostly concerns the OH substituents, limits the redox activity because it blocks the OH substituents, a major source of antioxidant actions. Therefore, methylated FLs establish a new mechanism of reactivity. For instance, isorhamnetin, a moderate scavenger in classical ABTS and FRAP tests, has been found to work efficiently during in vitro studies by inhibiting the production of ROS in LPS (lipopolysaccharide)-activated RAW 264.7 cells and blocking inflammation. In this trial, isorhamnetin was much more effective, than its parent compound, quercetin [38].

Sulfation is another modification of FLs, although naturally, only a few plant families produce this type of derivative [39]. The addition of a sulfate group incorporates a negatively charged fragment and increases the overall polarity of the entire molecule. Hence, a solid sample of sulfated FLs always contains counter ions, mainly sodium and potassium [40]. The most optimal for sulfation are the 3-OH and 7-OH substituents of AC moiety; however, the 3′-OH and 4′-OH of the B ring are also prone to this kind of reaction [39]. In general, sulfation is not required for the synthesis of artificial antioxidants. As deduced, the greater the number of sulfate substituents incorporated into flavonoid structure, the weaker the antioxidant that can be obtained.

The most common modification is glycosylation, and usually two types of bonds are formed during this reaction: the C- or O-glycosidic linkages, which further can yield α or β isomers [41]. O-glycosylation prevails in natural systems, and in this case, sugar attachment relies on the number and positions of OH substituents. The main sites are 3-OH and 7-OH of AC, although in some structures the 5-OH, 6-OH, 8-OH, or 4′-OH can also be glycosylated [42]. Other studies have linked the efficiency of O-glycosylation with the acidity of OHs, and considering this criterion, the reactivity decreases in the following order: 7-OH ≥ 4′-OH > 3-OH > 3′-OH > 5-OH [43]. Electrochemical studies carried out for quercetin and its glucoside derivatives confirmed that redox activity was decreased along with the number of attached sugar units [44].

In the case of C-glycosylation, it is hard to get a clear-cut answer on whether it is beneficial for the activity of FLs or not. For example, the C-glycosylated derivative of apigenin, 2″-O-β-D-xylosylvitexin, has been found to be less active in the ORAC assay than its parent compound or quercetin. However, the same molecule turned out to be more effective against hydroxyl and DPPH^•^ in in vitro studies with HepG2 (the human hepatocellular carcinoma cell line) and MCF-7 cells (the metastatic breast cancer cell line) [45]. In another study, the ability of compounds to scavenge DPPH^•^ decreased in the order apigenin < apigenin 6-C-glucoside-8-C-arabinoside < luteolin 6,8-di-C-glucoside< luteolin 6-C-glucoside < luteolin.

## 3. Metal–Flavonoid Interactions

FLs rich in π-electrons and bearing hydroxyphenyl and carbonyl groups can serve as electron donors and initiate bonding to a variety of metal ions [12]. The nature of the formed bond depends on the reactivity of metal ions and their electronic configuration. Two prevalent and most common metal–flavonoid interactions are coordination and redox interactions. Coordination bonds are typically formed by transition metal ions and main group metal ions with abundant vacant orbitals. The dominant force is the donation of an electron pair from a flavonoid molecule to an empty orbital of a metal ion. By contrast, redox interactions are produced by noble metals or by multivalent transition metals with proper reduction potentials. In this case, a donation of electrons provokes the reduction in metal ions to lower oxidation states or even to their metallic forms. Regardless of the type of interaction created, metal–flavonoid systems ensure a variety of dynamic and tunable properties, which are valuable to biochemistry and material science [12].

### 3.1. Metal–Flavonoid Compounds Formed via Coordination

Transition metal complexes are among the most frequently studied compounds owing to their ubiquitous role in biology and their versatile use in functional materials engineering. The donation of an electron pair from a ligand molecule to a metal ion ensures non-covalent bonding, which has several advantages over classical covalent bonds. Coordination bonds are characterized by higher dynamics, increased kinetic lability, and the ability to break, reform, or recreate [46]. In effect, the resulting complexes are thermodynamically less stable than molecules with traditional covalent bonding.

**Figure 4:**
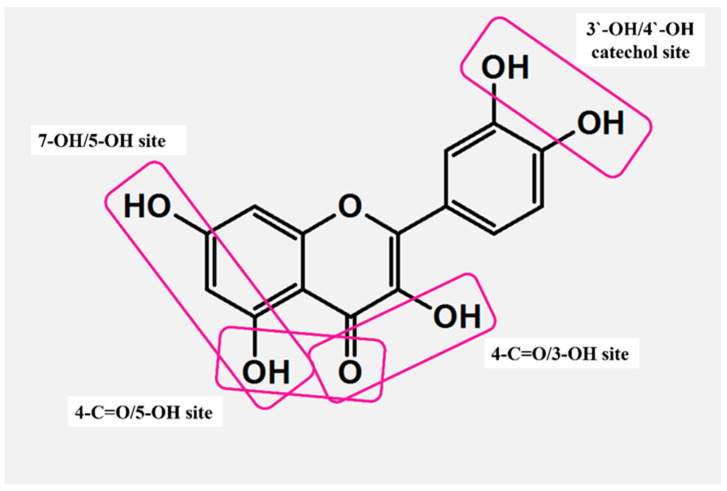
Active sites responsible for the formation of metal–flavonoid systems.

FLs contain several fragments that ensure coordination with transition metal ions (Figure 4). Among this group, three sites are the most effective: (i) 3-OH with 4-C=O from C, (ii) 5-OH from A with 4-C=O from C, and (iii) the catechol moiety (3′-OH and 4′-OH) from the B ring. More importantly, the coordination of FLs is usually pH-dependent. Due to the deprotonation of OHs, electron density around the donor changes, which makes the binding to metal ions more facile [47,48]. Acid–base equilibria studies revealed 3-OH from C as the strongest acid and 5-OH from A as the most resistant to deprotonation [47].

#### 3.1.1. Cu^II^ Complexes

**Figure 5:**
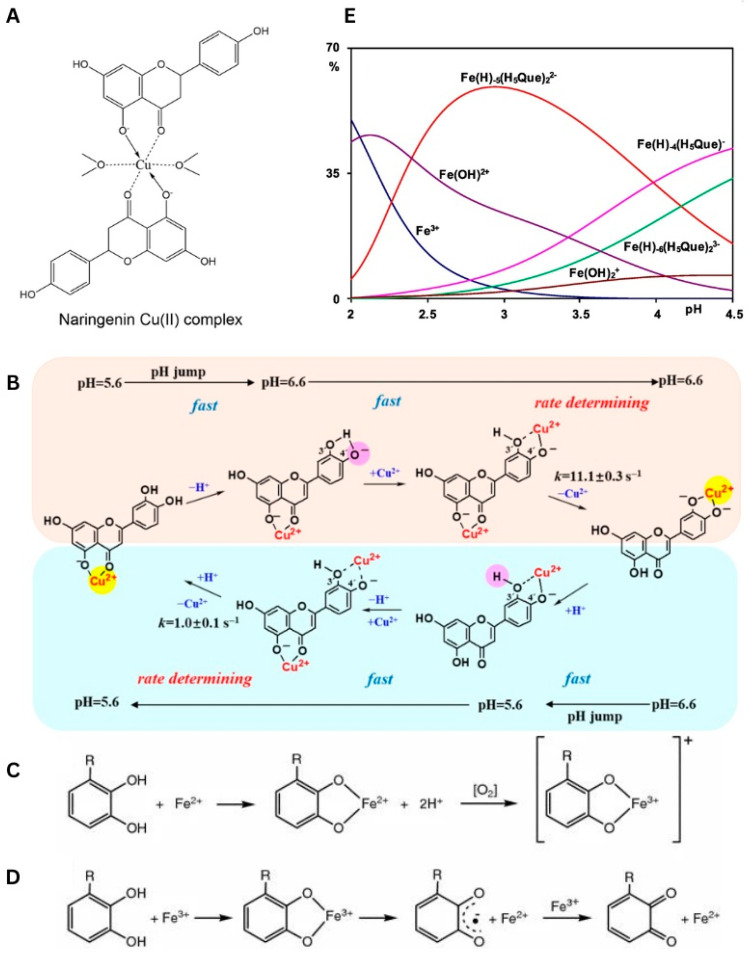
(**A**) Chemical structure of naringenin Cu(II) complex (solid state) [49]. Used with permission of Springer Nature BV, from [49]; permission conveyed through Copyright Clearance Center, Inc., Danvers, MA, USA. (**B**) Mechanism for Cu^II^ translocation in Cu^II^−Lut complexes resulting from changing from 5.6 to 6.6 and pH changing from 6.6 to 5.6 [52]. Reprinted (adapted) with permission from [52]. Copyright 2020 American Chemical Society. (**C**) Coordination of Fe^2+^ by polyphenols and subsequent electron transfer reaction in presence of oxygen generating the Fe^3+^−polyphenol complex. Used with permission from Springer Nature BV, from [53]; permission conveyed through Copyright Clearance Center, Inc. (**D**) Coordination of Fe^3+^ by polyphenols, subsequent iron reduction and semiquinone formation, and reduction in Fe^3+^ to form quinone species and Fe^2+^. R=H, OH [53]. Used with permission from Springer Nature BV, from [53]; permission conveyed through Copyright Clearance Center, Inc. (**E**) Distribution diagrams in presence of H_5_Que of Fe^III^ (C_M_ = 0.5 mM and C_L_ = 0.7 mM) [54]. Used with permission from Elsevier Science & Technology Journals, from [54]; permission conveyed through Copyright Clearance Center, Inc.

FLs bearing multiple OHs afford complexes with diverse structures, in which the mode and efficiency of coordination with Cu^II^ are directed by the number and location of O donors (Table S1). Starting from one of the simpler ligands, naringenin (Figure 1), its Cu^II^ complex was obtained in the solid state, and a physicochemical analysis was performed in both solid (X-ray, IR, and EPR) and solution (UV-Vis, NMR, and EPR) phases. In the solution phase, Cu^II^ was coordinated via the O ligands in a nearly square planar configuration (dx2-y2 ground state), and the binding was conducted involving 4-C=O (ring C) and 5-OH (ring A) donors. The 4-C=O/5-OH coordination introduced a new transition (charge transfer, CT) in the UV-Vis spectra and provoked variations in the signals of benzoyl moiety (‘band II’ from the π→π* transition). In crystalline form, Cu^II^ was bound by two anions of naringenin in the trans configuration (Figure 5A), and this bonding was completed by two molecules of secondary ligands, like water or DMF [49]. The dominance of 4-C=O/5-OH in Cu^II^ chelation was also confirmed in research by Tamayo et al., in which identical coordination was achieved in the presence of co-ligand 1,10-phenanthroline [50]. The same bonding was also achieved in the complex of apigenin, a molecule that has the same number and location of OHs, but in addition has a chromene unit. As evidenced, the replacement of a chromane fragment with chromene was not that critical for the binding to Cu^II^ [51].

A novel binding was revealed for luteolin (Figure 1), which possessed an additional OH substituent in the B ring. The structure of luteolin provides two fragments that are highly effective for coordination, namely 4-C=O/5-OH and 3′-OH/4′-OH (catechol moiety). Unlike in the previous cases, the binding of luteolin to Cu^II^ was pH-dependent, although conclusions presented on this issue differ depending on the technique used in studies. For instance, Malacaria et al. reported the formation of a complex already at pH = 4 based on data from potentiometric titration, which enables the detection of small portions of compounds, even at 100 µM concentration [55]. Slightly different results were presented by Xu et al., who claimed that the coordination was detected in the pH range of <5.0, 5.8> by applying UV-Vis absorption spectroscopy [52]. In both reports, the complexes were formed through a 1:1 M:L (metal:ligand) molar stoichiometry, and this element was not affected by the pH. Moreover, Malacaria et al. postulated the formation of two equally populated complexes, suggesting that both 4-C=O/5-OH and catechol sites are equally capable of binding to Cu^II^ [55]. In contrast, Xu et al. reported the pH-driven translocation of the metal ion. In this latter model, a change in conditions from acidic (pH < 6) to neutral, induces the deprotonation of 4′-OH. Further, this reaction provoked the dissociation of Cu^II^ from 4-C=O/5-OH and its binding by a catechol fragment. In reverse, a decrease in pH initiated the protonation of 4′-OH, thus enabling another switch of coordination from 3′-OH/4′-OH to 4-C=O/5-OH (Figure 5B). All these equilibria proceeded through the binuclear intermediate species [52].

A study of another flavonoid, kaempferol (Figure 1), showed that further hydroxylation at the third position of the C ring generates a new site capable of competing with the 4-C=O/5-OH fragment. The Cu^II^–kaempferol interaction was studied in a solution by applying EPR and UV-Vis spectroscopies [22]. As observed, coordination in such a system can proceed in two different ways, depending on the type of solvent used. In the dimethyl sulfoxide (DMSO) solution, Cu^II^ was coordinated mainly by 4-C=O/5-OH, while in methanol, the most favored was the 4-C=O/3-OH site. The advantage of this latter binding was also confirmed by applying DFT calculations [56].

By far, the most frequently studied flavonoid is quercetin (Figure 1), mainly because this molecule contains all three major coordination sites. A multispectroscopic UV-Vis/IR/NMR analysis in methanol revealed the formation of two diverse complexes, mononuclear and binuclear. The first included binding via the 3-OH/4-C=O moiety, which was independently confirmed by the DFT and the TDDFT (Time-Dependent Density Functional Theory). In turn, the coordination of the second involved two major sites simultaneously, that is 3-OH/4-C=O and catechol [57]. Different observations were delivered in the report by Torreggiani et al., in which the interaction with Cu^II^ was studied in water [58]. In mononuclear species, the addition of metal ions caused a strong decrease in the intensity of band I in the UV-Vis spectra of quercetin. Such a result suggested variations in the charge density of the B ring and indicated its catechol part as involved in coordination. As regards the binuclear complex, the binding to Cu^II^ provoked concurrent variations in the UV-Vis signals of benzoyl and cinamonyl parts. Therefore, the coordination mediated by catechol and 5-OH/4-C=O was deduced; however, the former was proposed as the primary binding site [58]. The most extensive investigation on interaction with quercetin was presented recently by Zhang et al., and in this work, the coordination was studied considering deprotonation equilibria. The molecule of quercetin contains five phenol moieties capable of losing a proton in the following order: 4′-OH, 7-OH, 3-OH, 5-OH, and 3′-OH, with the first deprotonation below pH 5.9. Interestingly, at pH = 5 and an M:L ratio below 0.0028, Cu^II^ binds primarily to the catechol fragment, while an increase in its concentration changes this binding by promoting the second 3-OH/4-C=O site [59].

#### 3.1.2. Fe^III^ Complexes

Unlike copper ions, interactions with iron ions do not always end with coordination. Instead, most FLs catalyze the oxidation of Fe^II^ to Fe^III^ and enhance this latter form (Figure 5C,D) [53]. This section includes ligands capable of forming classical coordination bonds, while the next chapter characterizes FLs involved in redox reactions (Table S2).

As observed, the coordination of apigenin results in the formation of stable chelate rings with the same binding pattern [15]. The Fe^III^ complex was produced at pH = 2 and with a 1:1 metal:ligand molar ratio, and this binding was tracked by monitoring Ligand-to-Fe^III^-Charge Transfer (LMCT) transitions (λ_max_= 520 nm) [60]. In the UV region, the coordination provoked a bathochromic shift of benzoyl and cinnamoyl bands. The positive charge of Fe^III^ interfered with the electron density of the ligand, causing an extension of π→π* transitions and shifting their bands to the visible part of the spectrum. Consequently, binding to Fe^III^ caused discoloration of the apigenin sample [61].

Among the listed FLs, luteolin (Figure 1) is the first that contains two competitive coordination sites, namely 5-OH/4-C=O and 3,4-catechol. Studies in ethanol showed that binding to Fe^III^ may proceed by establishing the equilibria at three different M:L stoichiometries: 1:1, 2:1, and 3:1. Furthermore, titration and ESI-MS experiments revealed that the equimolar ratio was the most effective in the production of a complex and the most stable species were produced in the binding of the 5-OH/4-C=O site. The addition of Fe^III^ changed the spectrum of luteolin by shifting the cinnamoyl and benzoyl bands to longer wavelengths [62]. The same effect was observed by testing the binding in water at pH = 6.5. However, in this latter case, a new band from π→d_π_ LMCT transitions was also reported [61]. As before, the changes in π→π* transitions explained the discoloration of the luteolin sample. Regardless of the solvent used, both studies proposed the catechol group as the main Fe^III^ binding site [61,62].

Another comparison was provided by using kaempferol (Figure 1) as a ligand. This time the priority was tested among the 5-OH/4-C=O and 3-OH/4-C=O fragments. Dimitrić Marković et al. showed that Fe^III^ binds to kaempferol under acidic (pH = 4.00) or weakly basic (pH = 8.00) conditions producing only an equimolar species. Further computational analysis revealed the dominance of the 3-OH/4-C=O site and the same result was also gained by collecting UV-Vis spectra [63]. In general, binding to Fe^III^ reduced the energy of the cinnamoyl and benzoyl bands by elongating the π-π system, and once again due to the coordination, the flavonoid sample became discolored [61].

### 3.2. Metal–Flavonoid Compounds Formed via Redox Interactions

The donation of electrons from FLs may also be irreversible, and instead of coordination, provoke the reduction in transition metal ions. The kinetics and the results of such a reaction are determined by the individual redox potential (E_1/2_). The greater the difference in E_1/2_ values, the easier will be the transfer of an electron in the redox pair. Compounds demonstrating high E_1/2_ can work as oxidants, while reagents with lower E_1/2_ usually donate electrons and play the role of reductants.

Particularly meaningful are reactions of FLs with multivalent transition metal ions, like Fe^III^ or Cu^II^. Beyond that, they are often responsible for prooxidative and inflammatory effects. The standard redox potential of free Cu^II^/Cu^I^ and Fe^III^/Fe^II^ are 0.153 and 0.77 V, respectively, and these values are modified by incorporating different types of ligands [64]. The interaction of FLs with complexes of Cu^II^ or Fe^III^ may follow two scenarios. During the first, the pro-oxidative species of Cu^I^ and Fe^II^ are produced through one-electron reduction. Further, free or loosely bound metal ions may provoke the Fenton and Haber–Weiss reactions and initiate the uncontrolled formation of ROS. Depending on physiological state, this overproduction can be quenched during the early stages. However, if all of the defense systems fail, the non-stop production of radicals will cause irreversible oxidative damage, inflammation, and cell death.

The second scenario assumes that the reduction in Fe^III^ and Cu^II^ may lead to metallic forms. This effect deserves broader discussion. It has been confirmed that some FLs are able to cross the blood–brain barrier and react in the central nervous system, where metal ions are abundant. Metal ion dyshomeostasis is one of the most common pathological features in neurodegeneration, and recently, nanoscale deposits of metallic Cu^0^ and magnetic Fe^0^ have been discovered in brain tissues isolated from subjects with Alzheimer’s disease (AD) [65]. Other studies have shown that FLs are able to interact with several AD and PD (Parkinson’s disease) targets, block the production of ROS (Reactive Oxygen Species), and restore redox balance [66]. Although nutrition rich in FLs is recommended to prevent inflammation, the use of phytochemicals in the treatment of AD and PD is a new paradigm and requires deeper investigation. As evidenced, the interaction of FLs with Fe^III^ and Cu^II^ is full of different dimensions, and in some cases, the dominant force is coordination in other redox reactions.

#### 3.2.1. Reactions with Cu^I^/Cu^II^ Ions

A systematic analysis of 24 different FLs revealed a reduction in Cu^II^ under four physiologically relevant conditions, that is at a pH equaling 4.50, 5.50 (established with an acetate buffer), 6.80, and 7.50 (achieved with HEPES or Tris-HCl buffers). The production of Cu^I^ was tracked by applying a selective BCS ligand and this production took place in two different pathways. In some cases, this process was gradual, and in others the production of Cu^I^ rose up to a certain point, after which a decrease in efficiency was observed. Naringenin, apigenin, and catechin isomers followed the first pathway, especially when the pH was higher. In contrast, other FLs like luteolin, kaempferol, and quercetin were effective only up to some point, and in this case, the pH had no effect [67].

Independently, the bathocuproine assay, performed at pH = 7.50, revealed a reduction in Cu^II^ catalyzed by catechin and epicatechin at an M:L 2:1 ratio [68]. Likewise, using the same method, the reducing properties were confirmed for kaempferol and quercetin, even at a stoichiometry of M:L 3:1 [69,70]. Above all, compounds bearing catechol moiety were the most effective in the reduction. In contrast, such a reaction was inhibited by ligands containing the C2-C3 double bond and 4-C=O in the C ring [67,68,70]. Up to now, the only data about the coordination of reduced copper were presented for quercetin. The equimolar complex was produced and metal chelation was achieved by engaging the 3-OH/4-C=O site. Binding to Cu^II^ changed the distribution of a charge, thus the complex was found to elongate the π-π system and shift the cinnamoyl and benzoyl bands on UV-Vis to the longer wavelengths [71].

#### 3.2.2. Reactions with Fe^II^/Fe^III^ Ions

The reaction in the iron–flavonoid pair depends mainly on the structure of the latter reagent and the conditions applied. For instance, naringenin binds Fe^III^, yielding a stable complex, but its incubation with Fe^II^ in water (pH = 7 or 6.5) or DMSO solution promotes immediate oxidation of the metal [61,72]. As a result, freshly formed Fe^III^ interacts with the flavonoid, forming a classical coordination bond via the 5-OH/4-C=O site [61]. Interesting results have also been observed for luteolin when the reaction with Fe^III^ was induced in hot water (90 °C). Such an approach was introduced to imitate nutrient intake from cooked edible plants, and in this case, an ESI-TOF MS analysis revealed the formation of two complexes, the major with Fe^III^-luteolin (M:L 1:2), and the minor with Fe^II^. The presence of the latter compound was confirmed additionally using a 1,10-phenanthroline indicator [73].

A reduction in Fe^III^ was also achieved using quercetin. Three independent ESI-MS studies revealed that mixing Fe^III^ with quercetin provides a prevalence of Fe^II^ complexes over Fe^III^ species [12,74,75]. Surprisingly, this effect was possible using different solvents, like 50 mM acetate buffer (pH = 5.50) or a methanol–water mixture (1:1). Most importantly, a reduction in Fe^III^ was possible owing to the proper distribution of electrons in the structure of the flavonoid. This proper distribution was provided by the conjugation between the 2,3-double bond and the 4-C=O group [15].

Horniblow et al. established that quercetin coordinates to Fe^II^ and Fe^III^ with binding constants of K= 8.30 × 10^−5^ M^−1^ and K= 3.86 × 10^−6^ M^−1^, respectively [76]. Similar data were presented in the work by Guo et al., in which the conditional binding constant for equimolar Fe^II^–quercetin was 10^−6^ − 10^−7^ M^−1^. Quercetin binds Fe^II^ rapidly (within~ 1 min) forming complexes at either a 1:1 or 1:2 M:L molar ratio. In turn, coordination with Fe^III^ was five times slower and generated not only an equimolar complex but also a quantity of Fe^II^ species [77,78]. As for Fe^II^–quercetin, a complex with 1:2 stoichiometry was identified as dominant based on the DFT and spectral data (UV-Vis, IR, ESI-MS, and NMR) [79]. Likewise, in the ^1^H NMR spectra, all ^1^H signals became shifted and broadened, suggesting the formation of a high-spin complex. The greatest variations were noticed for the 3-OH signal. Also, an independent CV analysis showed that binding to Fe^II^ affected the oxidation of this group [80]. Thus, it has been postulated that quercetin coordinates to Fe^II^ by engaging the 3-OH/4-C=O fragment. It has been suggested that the dominance of 3-OH/4-C=O over other parts was due to the stronger acidic nature of 3-OH and because of the weak iron-chelating properties of catechol [54,79].

Although incubation of Fe^III^ with quercetin primarily leads to a reduction in the former, coordination with this metal ion is possible under the right conditions. For instance, three different Fe^III^ complexes were detected in water within the pH range of 2.00–4.50. The first and most abundant complex, Fe(H)_−5_(H_5_Que)_2_^2−^ (M:L 1:2), was detected already at pH = 2.00, while the remaining Fe(H)_−4_(H_5_Que)^−^ (M:L 1:1) and Fe(H)_−6_(H_5_Que)_2_^3−^ (M:L 1:2) complexes emerged and coexisted around pH = 3.00. Together, all these species reached 30% of the total Fe^III^ population and decreased the concentration of other coappearing complexes, such as Fe(OH)_2_^+^ and Fe(OH)_2_^+^ (Figure 5E). Additionally, DFT studies revealed the binding of Fe^III^ to catechol moiety in the dominant Fe(H)_−5_(H_5_Que)_2_^2−^ form. Coordination with Fe^III^ can also be achieved in an aprotic solvent, like DMSO. However, unlike previous findings, only two main Fe^III^ species can be obtained under such conditions. Notably, the aprotic environment promotes coordination via the 3-OH/4-C=O site [54].

A partial reduction in Fe^III^ was also observed after the use of catechin. Catechin was proposed to support Fe^II^ more than Fe^III^ and inhibit its oxidation [81]. In the Fe^III^/Fe^II^–catechin mixture, two Fe^III^ complexes were formed with 1:1 and 1:2 M:L molar stoichiometries. In contrast, interaction with Fe^II^ yielded only one compound in an equimolar ratio [82,83]. Catechins exhibit a lower affinity towards Fe^III^ compared to bacterial siderophores, enterobactin, and desferrioxamine, and are comparable to the affinity provided by the commonly used chelators, NTA and hydroxamic acid [60,82]. As observed, binding to Fe^III^ in water (pH = 7.40) resulted in a new absorption band from the LMCT transition, and the energy of this signal indicated coordination by the catechol site [61,82]. Identical conclusions were presented by Porfírio et al. based on CV data [80].

## 4. Redox Activity of Metal–Flavonoid Systems

There is no one correct pathway in which metal–flavonoid systems mediate redox reactions. The first, and most studied, involves the synthesis of metal complexes and further analysis of their activity. The second pathway comes from spontaneous redox-based interactions. In this case, the electron-rich flavonoid affects the oxidation state of the metal ion, and vice versa. A common outcome of such in situ interaction is the generation of redox-stable forms [84]. This chapter describes the redox chemistry of Cu^II^ and Fe^III/II^ complexes with FLs. The first part collects information on pre-formed (synthesized) complexes, while the second presents the behavior of the in situ systems, that is, compounds created through spontaneous interactions.

### 4.1. Pre-Formed Metal–Flavonoid Complexes

**Figure 6:**
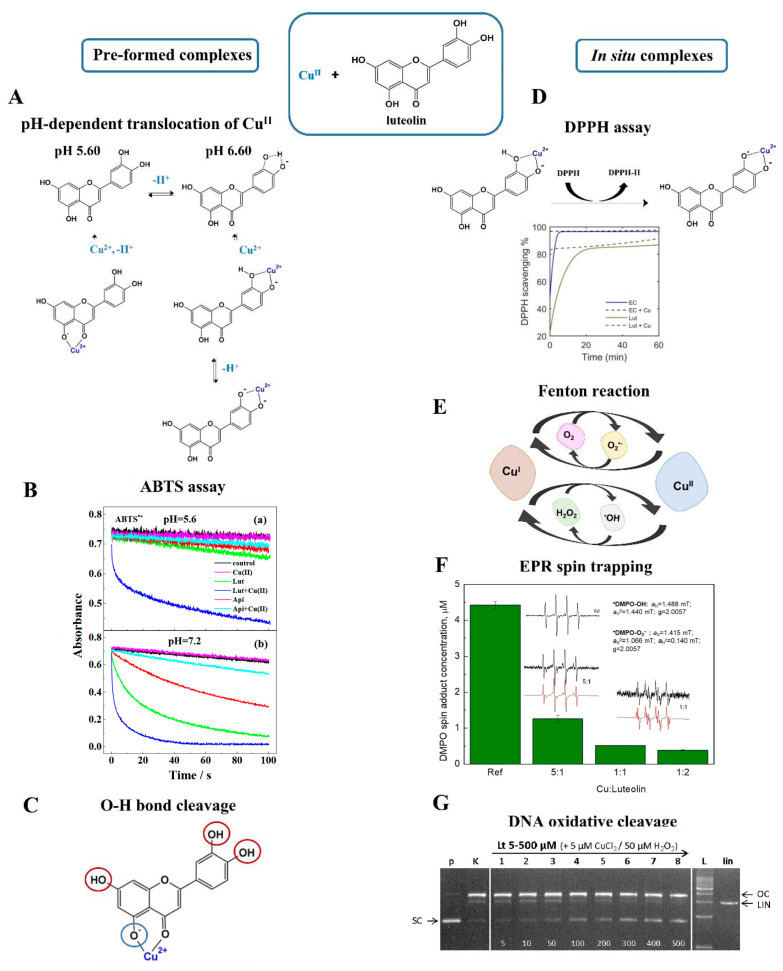
Schematic view on redox activity of pre-formed (left) and in situ (right) produced Cu^II^–luteolin complexes. (**A**) pH-dependent coordination and translocation of Cu^II^ between 5-OH/4-C=O and the 3′−OH/4′−OH sites based on work by Xu et al. [52] (**B**) Time-dependent reactivity of pre-formed Cu^II^−luteolin M:L 1:1 against ABTS^•+^ cation radicals. Upper plot (a): activity of species formed at pH 5.60; bottom plot (b): activity of species formed at pH of 7.20 [52]. Reproduced with permission from [52]. Copyright 2020, American Chemical Society. (**C**) Coordination with Cu^II^ affects the strength of O−H bonds. The impact of Cu^II^ was deduced based on DI parameters [51]. Red circles: faster proton cleavage. Blue circle: disturbed proton cleavage. (**D**) Time-dependent scavenging of DPPH^•^ radicals provided by in situ Cu^II^−luteolin [85]. Reproduced with permission from [85]. Copyright 2022, Lee and Heffern. (**E**) Copper-catalyzed Fenton reactions. (**F**) EPR spin-trapping performed for in situ Cu^II^−luteolin at different M:L molar ratios. DMPO was used as spin trap and H_2_O_2_ was applied to initiate Fenton reactions [86]. Reprinted from [86]. Copyright 2022, with permission from Elsevier. (**G**) Anti-Fenton activity of in situ Cu^II^–luteolin verified by the ROS−catalyzed DNA cleavage experiment [86]. Reprinted from [86]. Copyright 2022, with permission from Elsevier.

It is difficult to gain a clear-cut answer to how metal chelation affects the redox activity of FLs. On one side, coordination and radical scavenging are provided by the same moieties, therefore binding to metal ions may block the actions of certain fragments. On the other, the charge of metal ion affects the electron density of AC and BC segments, thus favoring the cleavage of protons during the HAT- or SPLET-type reactions. One of the most interesting behaviors was presented recently for the Cu^II^–luteolin complex, in which changes in pH provoked the translocation of metal ions. At a pH of 5.60, Cu^II^ was coordinated by the 5-OH/4-C=O site and the scavenging was mediated only by the catechol part (3′-OH/4′-OH). In contrast, the increase in pH to 6.60 provoked the migration of metal ions to the 3′-OH/4′-OH fragment thereby enhancing the antioxidant potential of 5-OH (Figure 6A). Cu^II^–luteolin was proven to be effective against ABTS^•+^ cation radicals in two independent studies, but more detailed information on electron distribution was gained based on Barder’s QTAIM (Quantum Theory of Atoms in Molecules) analysis (Figure 6B) [51,52]. As evidenced, most of the unpaired electrons were located at the Cu^II^ center. In addition, the DI (delocalization index) parameters reported a reduction in the strength of almost all O-H bonds, except for 5-OH (Figure 6C). This impairment enabled a faster cleavage of protons, thus increasing the antioxidant potential of the complex. This last conclusion was partially coherent with the values of BDE, PA (proton affinity), and ETE (electron transfer enthalpy) parameters. Significant changes were observed in the distribution of electrons upon the binding of Cu^II^ to 5-OH/4-C=O. Specifically, the dissociation of protons from catechol and 5-OH was promoted while the dissociation from 7-OH was disturbed [51]. Coordination with Fe^III^ did not result in the formation of such an effective scavenger, as with Cu^II^. Fe^III^–luteolin delivered lower antioxidant activity in DPPH, ABTS, and FRAP tests than the ligand and these results were consistent with the data gained from CV [55,62]. The binding of Fe^III^ provoked a shifting of CV signals towards a higher potential and their greater separation. This, in consequence, indicated a slower transfer of electrons and weaker redox activity [62]. Further analysis revealed an increase in the value of PA parameters which explained a moderate involvement of Fe^III^–luteolin in HAT- and SPLET-based reactions. The weaker activity of Fe^III^–luteolin against radicals suggested slightly different electron distribution and the formation of a redox-stable form [55].

Coordination with Cu^II^ turned out to be beneficial for the activity of another flavonoid, kaempferol. Cu^II^–kaempferol showed significantly higher activity in the ABTS assays than the parent ligand [22,51]. Similarly to Cu^II^–luteolin, the chelation of Cu^II^ affected the electron density in flavonoid molecules, thus changing the strength of bonds in all OH substituents. Further DFT analysis revealed a decrease in the BDE parameters of 3-OH and 5-OH, suggesting an involvement of these parts in HAT and SPLET reactions. On the contrary, the cleavage of protons was suppressed for the 4′OH and 7-OH [51]. The synthesis of the Cu^II^–kaempferol was favorable for direct scavenging, as the reaction with DPPH^•^ was much more effective when the complex was applied. As deduced, coordination with Cu^II^ promoted the formation of semiquinone radicals, thus increasing the antioxidant potential of the flavonoid [85]. Completely different were the results gained for Fe^III^–kaempferol. Preliminary studies showed that the presence of Fe^III^ reduced reactivity against DPPH^•^ [63]. However, a detailed explanation of how the Fe^III^ chelation affects the activity of kaempferol is still lacking.

By far the most studied were the complexes of quercetin. In standard DPPH and ABTS assays, Cu^II^–quercetin showed higher scavenging activity than quercetin [87]. The mechanism assumed the homolytic cleavage in the bond of 4′-OH and the formation of a semiquinone radical. This product was stabilized by two elements, the charge of metal iona and the conjugation with 3-OH. Apart from this, the antioxidant potential of Cu^II^–quercetin was directed by the relation between the energy of Cu^II^–semiquinone and Cu^I^–quercetin. Notably, the formation of stable Cu^I^–quercetin reduced the potential of Cu^II^–quercetin, and in reverse higher activity was promoted by stable Cu^II^–semiquinone [88].

Much fewer data were gained for the pre-formed Fe^III^–quercetin, mainly because quercetin catalyzed the reduction in Fe^III^. The formation of a stable Fe^III^ species is possible, but only under radically acidic conditions, and this in turn impedes the performance of DPPH and ABTS assays [54]. Hence, Fe^II^–quercetin species are usually involved in redox studies. Fe^II^–quercetin displayed a greater reactivity against DPPH^•^ than the free flavonoid [89]. CV measurements performed in a phosphate buffer (a pH of 7.40) confirmed the ability of the complex to undergo fast and effective oxidation. Moreover, it was shown that Fe^II^ chelation encouraged the formation of a stable semiquinone radical, thereby increasing the final redox activity [80,89].

### 4.2. In Situ Metal–Flavonoid Systems

Given the metal–flavonoid systems, it seems unlikely that pre-formed complexes are the only species established in a natural environment. It is more probable that FLs interact with metal ions more spontaneously, yielding in situ forms. In addition, due to diverse electron distribution, in situ complexes may demonstrate completely different properties than their pre-formed counterparts. Interesting observations were presented for the in situ interaction of luteolin with Cu^II^. Free luteolin was responsible for the scavenging of more than 80% of DPPH^•^, but this effect was even greater when free Cu^II^ ions were added to the solution (Figure 6D) [85]. The main question was whether this effect was caused by the redox-active metal ion itself or rather its presence and charge affected the activity of luteolin. The correct answer was provided by further experiments, in which Cu^II^ was replaced by redox-inert Zn^II^. This trial shared the same trend, namely much more efficient scavenging of DPPH^•^ in the presence of the metal ion, indicating that the redox activity of the inorganic component had no effect. The improved actions of the in situ-formed complexes were observed also in the ABTS assay. The presence of either free Cu^II^ or free luteolin was not enough for the complete quenching of radicals, but this aim was achieved when the reagents were applied together [67].

Free Cu^II^ influenced the reactivity of luteolin, but the fact remains that this effect works both ways, and the flavonoid can also affect the activity of the metal ion. The coumarin-3-carboxylic assay (3-CCA) showed that the presence of luteolin blocked the involvement of free Cu^II^ in Fenton reactions (Figure 6E) [85]. Similar conclusions were gained from an EPR spin-trapping experiment, in which the in situ formed Cu^II^–luteolin suppressed the production of two major ROS, hydroxyl radical and superoxide radical anion, up to 80% (Figure 6F). More importantly, such a result was gained by using a DMSO-H_2_O mixture in the presence of atmospheric O_2_, namely redox-active chemicals typically involved in radical reactions [67]. An immediate inhibition of Fenton reactions was also accomplished after the addition of kaempferol. In this latter case, the EPR spin-trapping experiments revealed the greatest effect when Cu^II^ and flavonoids were mixed in an equimolar ratio [22].

Luteolin also demonstrated its anti-Fenton actions in experiments with plasmid DNA. Typically, free Cu^II^ mixed with H_2_O_2_ or ascorbic acid promotes the production of ROS, which further initiates cleavage and oxidative damage in the structure of plasmid DNA (Figure 6G). The in situ interaction with luteolin has been found to hamper these reactions. Surprisingly, luteolin provided a much greater anti-Fenton effect compared to other FLs like kaempferol or quercetin, and even a fourfold excess of these latter reagents was not efficient for protection against DNA cleavage [22,67,70].

One of the most critical pieces of data on the activity of in situ complexes was delivered by Lewis et al. [84]. In this case, the activities of quercetin, Cu^II^, Fe^III^, and their pre-formed and in situ complexes were investigated against the O_2_^•−^/O_2_ redox pair. As demonstrated with CV, free quercetin successfully captured both O_2_^•−^ and O_2_, yielding a decrease in their anodic and cathodic signals. In contrast, free metal ions had a negligible effect on both oxygen reagents, and only Cu^II^ slightly reduced the concentration of O_2_ by provoking its reaction with Cu^I^. Significantly improved were the results gained for the in situ complexes. For instance, Fe^III^–quercetin formed at a molar ratio of 1:1 (M:L) caused a decrease in anodic and cathodic signals, and this result was even greater when the flavonoid was added in double excess. Likewise, the greatest quenching of the O_2_^•−^/O_2_ redox pair was provided by Cu^II^–quercetin formed in situ at a 2:1 molar ratio. In general, the in situ Fe^III^ and Cu^II^ complexes were less active against molecular O_2_ than free quercetin and were comparably active towards the O_2_^•−^ radical. Furthermore, clear differences in the behavior of the in situ compounds and their pre-formed counterparts were revealed. The synthesized Fe^III^–quercetin was found to be less active against both oxygen chemicals. By contrast, the pre-formed Cu^II^–quercetin exhibited analogical activity as the free ligand only against the O_2_^•−^ radical [84].

## 5. Metal–Flavonoid Materials

FLs can be exploited as building blocks in the preparation of metal–phenolic networks (MPNs), and they attract considerable interest in this field because of their structural diversity and multiple coordination sites. Site-selective coordination enables the engineering of MPNs with a high degree of functionality [8]. In addition, depending on the structure and the type of attached substituents, most FLs catalyze redox transformations of metal ions, thus providing an alternative mechanism for MPN production. Hybrid metal–flavonoid materials are usually formed by employing coordination- or redox-driven assembly.

### 5.1. Coordination-Driven Construction of Materials

**Figure 7:**
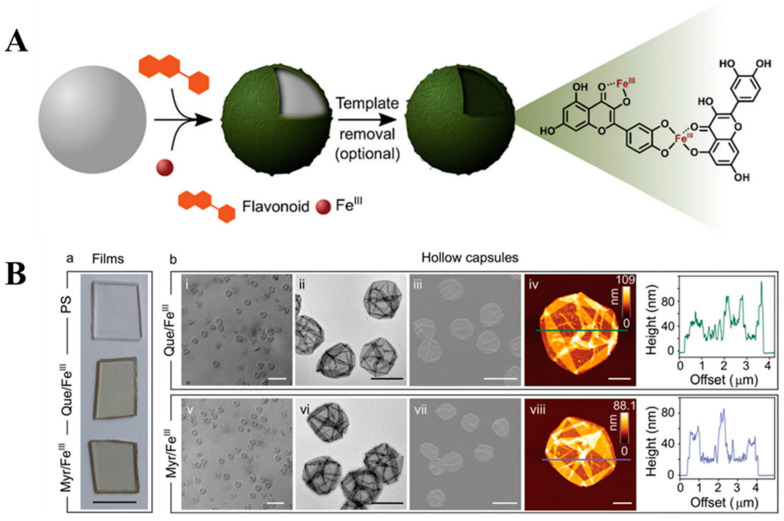
(**A**) A schematic view on the concept of the coordination-driven assembly of MPNs on a templating surface. Fe^III^ and quercetin were used to mediate the assembly. The network was established involving three different coordination sites [8]. Used with permission from the Royal Society of Chemistry, from [8]; permission conveyed through the Copyright Clearance Center, Inc. (**B**) Characterization of MPN films (a); PSs are the uncoated particular substrates, and the remaining systems are the films prepared from Fe^III^–quercetin and Fe^III^–myricetin. From left to right: differential interference contrast (DIC) microscopy, transmission electron microscopy (TEM), scanning electron microscopy (SEM), and atomic force microscopy (AFM) with the height profile (b) [8]. Used with permission from the Royal Society of Chemistry, from [8]; permission conveyed through the Copyright Clearance Center, Inc. FLs with multiple coordination sites enable the construction of MPNs by engaging different parts of the structures. Moreover, the control over coordination gives the possibility of constructing materials with high precision of structure and functionality. Switching the binding between catechol, carbonyl, and hydroxyl groups delivers more flexible materials. In this way, the mechanical and physicochemical properties of MPNs can be easily tuned only by directing cross-linking. The synthesis of MPNs with an approach of site-selective coordination was investigated on FLs containing the 4-C=O group, namely quercetin, chrysin, 3-hydroxyflavone, and 3′,4′-dihydroxyflavone. All these ligands served as binding modulators and Fe^II^ and Fe^III^ ions were used to trigger coordination and cross-linking [8]. In general, the coordination with Fe^III^ was more favorable because of the production of thermodynamically stable systems.

Coordination-driven assembly of materials has primarily been explored using tannic acid, gallic acid, and epigallocatechin-3-O-gallate, yielding promising results with each of these compounds [90,91]. The implementation of FLs in this direction is still untapped, although a first step forward has already been taken by establishing the production of MPNs through coordination with Fe^III^ [8]. Four different FLs were tested in this approach, i.e., myricetin, quercetin, fisetin, and luteolin. Thin MPN films were specifically prepared using catechol-containing ligands, explaining the choice of reagents [8,90]. Mixing FLs with Fe^III^ in the presence of polystyrene particles (PS) resulted in immediate growth of colored films (Figure 7). After complexation, a strong bathochromic shift of the latter signals was observed, indicating that cross-linking in both systems was induced by the binding of catechol fragments. Removal of the PS caused the formation of hollow capsules (Figure 7B). Both the PS-bound films and the capsules displayed similar antiradical activity by consuming the same amount of DPPH^•^. Surprisingly, the Fe^III^–quercetin film demonstrated a 10% higher scavenging activity than free flavonoid, and the coordination with Fe^III^ was proven to stabilize its intermediate species. Additionally, the activity of Fe^III^–quercetin was maintained over many cycles. The reusability of MPNs, especially during multi-cycle reactions, and their rapid assembly ensured Fe^III^–quercetin a strong advantage over free quercetin [8].

The 3-OH/4-C=O of quercetin exhibited the highest affinity to Fe^II^. More specifically, it has been shown that under acidic conditions, Fe^II^ was mainly coordinated by 3-OH/4-C=O, while an increase in pH promoted its binding to the second site. The 3-OH/4-C=O of quercetin was also predominated in the coordination with Fe^III^, whereas 5-OH/4-C=O and catechol were less favorable in this case [14]. Therefore, according to reports, the construction of MPNs can be influenced by pH. Consequently, it is possible to obtain materials with different quercetin–Fe^II^ ratios, wall thicknesses, and binding modes. Synthesis conducted under alkaline conditions promoted the Fe–catechol binding, leading to increased cross-linking density and greater stability of the resulting materials. The type of coordination was also found to be decisive for other mechanical properties [14].

The pH- and coordination-driven assembly of MPN capsules was also explored using other FLs like luteolin and fisetin. Similar to the findings mentioned above, the physicochemical properties of the resulting materials depended on the conditions applied during the synthesis [14].

In this approach, the MPN films were prepared by mixing crude eucalyptus extract (Euc) containing four different FLs, quercetin, myricetin, gallocatechin, and catechin, and their glucoside derivatives, apigenin-7-glucoside and isorhamnetin-7-*O*-glucoside, with a Fe^III^ solution in the presence of solid substrates [11]. After the removal of a solid template, stable and monodisperse Euc/Fe^III^ capsules were formed. In terms of morphology, the spheres were rather collapsed, as observed on DIC (differential interference contrast microscopy) and AFM images. In addition, EDX (energy-dispersive X-ray spectroscopy) and XPS (X-ray photoelectron spectroscopy) analyses revealed an abundance of Fe^III^ oxidation state in their network, while the LMCT band at 565 nm indicated catechol-induced cross-linking.

The disassembly of Euc/Fe^III^ capsules released only two phenolic components: myricetin and quercetin, in concentrations of 0.33 and 0.04 pg per capsule, respectively. Interestingly, these two FLs were involved in MPN formation, despite their low concentrations in the initial Euc extract (67.4 and 20.3 µg/mL, respectively), suggesting high selectivity in cross-linking. Independent studies conducted on model mixtures include the following: myricetin/gallocatechin/catechin and epigallocatechin gallate/catechin/gallocatechin. These indicated that only compounds bearing the catechol fragment were typically involved in the synthesis of MPNs [11]. The dominance of catechol-containing regents was also probed by applying a mathematical simulation. The calculations based on the random walk model showed that over the time of film growth, FLs with ‘single-chelating-site’ lost their contribution to metal binding, while the participation of compounds with ‘two-chelating-sites’ increased. Apart from this, the use of different solid platforms did not affect the composition of the final products. As evidenced, myricetin and quercetin were the main components of Euc/Fe^III^ films prepared on PMMA (poly(methyl methacrylate)), mesoporous silica, aminated silica particles, or planar quartz substrates. Nevertheless, the type of surface turned out to be decisive for the final myricetin–quercetin ratio in the network. The Euc/Fe^III^ films demonstrated slightly improved antioxidant activity against DPPH^•^ compared to a simple mixture of free FLs. Moreover, the MPNs maintained their antiradical properties during the multiple cycles, enabling their application over a longer period [11].

**Figure 8:**
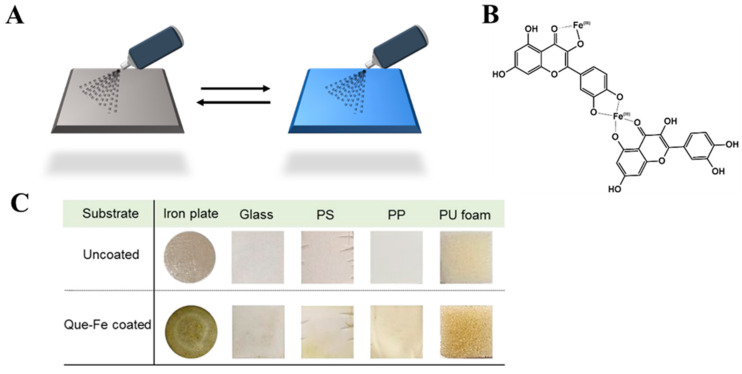
(**A**) The preparation of MPNs using the spray-assembly method. Solutions of FLs and metal ions are alternately sprayed onto a solid substrate [92]. Reproduced with permission [92]. Copyright 2018, American Chemical Society. (**B**) Coordination sites involved in the assembly of MPNs. (**C**) The characterization of Fe^III^–quercetin films prepared onto various substrates: iron plates, glass, polystyrene (PS), polypropylene (PP), and polyurethane (PU) [92]. Reproduced with permission [92]. Copyright 2018, American Chemical Society.

The synthesis of MPN films through the simple mixing of reagents can be successfully applied to small and even microscopic objects. However, adapting such a reaction to an industrial scale, where larger and uneven surfaces are usually used, poses a greater challenge. A spray-based assembly was developed to solve this issue by upgrading the fabrication of MPN coatings and adjusting it to a variety of sizes and shapes (Figure 8A). The films were made from Fe^III^, quercetin (Figure 8B), or other phenolic ligands like tannic acid, gallic acid, or multiligand systems. The coatings were tested on several objects like metallic Fe, SiO_2_, polystyrene, polypropylene, and polyurethane (Figure 8C). To achieve cross-linking, each of these objects was covered with two layers: a primer with a ligand or ligands mixture and a second with a Fe^III^ solution. As deduced, the thickness of the MPN depended on the size and structure of the flavonoid used, further allowing for customization of this method [92].

### 5.2. Redox-Promoted Construction of Materials

The synthesis of MPNs can also be preceded by redox reactions. In this case, the conventional metal salt solutions can be replaced by using metallic objects, like rusted iron nails [10,93]. The etching of the rust materials, earlier immersed in the flavonoid extract, provoked the binding between oxidized metals and FLs. This reaction resulted in complexes capable of growing films on the dispersed solid substrates. As regards thermodynamics, the rust-mediated approach expands the field of interfacial chemistry by incorporating the double-dynamic etching step with the self-assembly of materials. An important feature of this procedure is its predictability and simplicity. This contrasts with the conventional preparation of MPNs from salt solutions, which typically tends to be more chaotic. As observed, the rust-mediated synthesis enabled facile control over the material thickness only by adjusting the time of the reaction. For example, the cross-linking conducted under a minute yielded materials with a thickness of 10 nm, and this number was gained regardless of the applied components and conditions [90,94].

**Figure 9:**
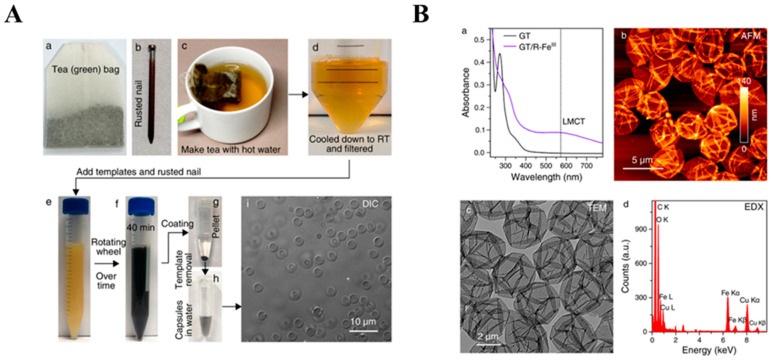
(**A**) The procedure of the preparation of MPNs using a green tea (GT) infusion (a) as the source of flavonoids and rusted nails (b) as the source of metal ions. The GT infusion was prepared in hot water (c), followed by filtration and cooling to room temperature (d). PMMA microparticles were added to the GT infusion to serve as a solid template. Rusted nails were incubated in the GT infusion to initiate the assembly of Fe^III^-GT networks (e–h). The DIC image (i) confirmed the formation of hollow capsules after template removal [93]. Reproduced with permission [93]. Copyright 2018, American Chemical Society. (**B**) The characterization of Fe^III^-GT capsules. Top-left (a): UV-Vis absorption spectra of the GT infusion and Fe^III^-GT capsules suspension. Top-right (b): AFM image. Bottom-left (c): TEM image. Bottom-right (d): EDX spectrum confirming the coordination with Fe^III^ in the formed MPN [93]. Reproduced with permission [93]. Copyright 2018, American Chemical Society.

The rust-mediated assembly was initially studied using pure ligand solutions. However, the success of this method has encouraged its exploration also through the use of crude tea infusions (Figure 9) [10,93]. The chelation of Fe^III^ from a rusted nail by green tea FLs, mainly catechins (GTC, green tea catechins), turned the color of the initial dispersion into blue-black, confirming the complexation through catechol and galloyl sites (Figure 9A). The efficiency of the etching was tracked using UV-Vis, and the LMCT band at ~570 nm confirmed the coordination of Fe^III^ by GTC (Figure 9B). The signal intensity increased continuously for 3 h until reaching a plateau and revealing completed self-assembly. The cross-linking triggered the production of films on the particle templates. The removal of solid substrates resulted in the conversion of the formed materials into hollow capsules, although this process succeeded only for samples fabricated for a longer time (Figure 9B). For the remaining systems, differential interference contrast (DIC) microscopy revealed the presence of colloidally stable and monodisperse Fe^III^-GTC spheres, while the conventional solution-based methods yielded polydispersion and aggregation of capsules. The disassembly of MPNs in concentrated HCl led to the release of epigallocatechin gallate and epigallocatechin. Thus, the rust-mediated assembly was concluded to ensure selectivity by promoting the binding of only appropriate FLs [93].

## 6. Outlook and Conclusions

The fabrication of synthetic materials has changed dramatically in the last decade. Currently, more and more attention is paid to chemicals of plant origin and procedures that align with the principles of green chemistry. Additionally, much effort is still dedicated to the production of complex, but functional artificial materials. There is a particular need to develop sophisticated systems that can keep up with the heyday of nano- and biotechnologies.

The challenge is, therefore, to transform its structural and functional perfection into artificial systems relying mostly on the character of individual reagents. This Review places a special emphasis on hybrid inorganic–organic materials established from plant-derived FLs and transition metal ions.

The self-assembly of metal–flavonoid materials can be directed by choosing an appropriate inorganic and organic component. In addition, the formation of supramolecules can be site-selective and pH-dependent, and this enables greater control over the structure and behavior of a final product. In simple metal–flavonoid complexes, the coordination is selective and depends on the type of metal ion and the structure of the ligand, and this property is maintained also in the macroscale during the self-assembly of networks. In this way, studies on simple and small models can be converted to higher systems in order to predict the structure and behavior of components in more advanced structures. Equally important is the upgrade of functionality. Often, metal–flavonoid materials have been found to be more effective than small and simple complexes formed by the same chemicals.

Understanding and mastering the principles of metal–flavonoid interaction is essential to create functional materials. Control over the structure, hierarchy, and sequence of building blocks may provide precision in the design of man-made materials. Such a strategy is attainable since the assembly of networks is fully guided by site-selective coordination. Furthermore, the construction of sequence-defined materials enables the shaping of interactions between components, and by that, it is possible to achieve proper folding, stiffness, and toughness. Control over intermolecular forces gives an opportunity to develop complex systems with desired mechanical properties.

Significant achievements have been attained in the engineering of metal–flavonoid materials in the last few years. Still, there are many unresolved issues. Some of the basic questions are essential also for the idea of AI-aided synthesis planning. In this area, one of the missing points is the impact of transition metal ions. Still, it needs to be explored thoroughly how properties of metal ions such as size, charge, valence state, electron configuration, and reduction potential affect the cross-linking and assembly of materials. Further research should also include flavonoid ligands. In this case, the most important is to verify the role of selected OH substituents and their acid–base properties. Considering the kinetics and dynamics of self-assembly, it is worth investigating the influence of environmental factors like temperature, pH, ionic strength, solvent type, and the presence of co-ligands.

Summing up, a comprehensive analysis of metal–flavonoid systems will provide further advances in the construction of sophisticated materials. Computer-aided planning will meet the expectations of modern technologies by developing complex functional matter. Moreover, synthesis supported by artificial intelligence will be helpful in mimicking nature, enabling the assembly of hybrid inorganic–organic macromolecules, and ensuring high precision of structure and functionality.

## Data Availability

The data presented in this study are available on request from the corresponding author.

## Supplementary Materials

The following supporting information can be downloaded at: https://www.mdpi.com/article/10.3390/molecules29112573/s1, Table S1: Structural and physicochemical details on Cu^II^/Cu^I^–flavonoid systems established by coordination or redox interactions. Table S2: Structural and physicochemical details on Fe^III^/Fe^II^–flavonoid systems established by coordination or redox interactions.
