# Supplementary material for: Metal–Flavonoid Interactions—From Simple Complexes to Advanced Systems
Source: Molecules. 2024 May 30;29(11):2573. doi: 10.3390/molecules29112573 (PMC11173564; doi:10.3390/molecules29112573)
Supplement: Supplementary file 1 [file molecules-29-02573-s001.zip › molecules-2973956-supplementary.pdf]

molecules

MDPI

Supplementary

Metal–Flavonoid Interactions—From Simple Complexes to Advanced Systems

Paulina Katarzyna Walencik [1,*], Renata Choińska [1], Ewelina Gołębiewska [2] and Monika Kalinowska [2,*]

[1] Institute of Agricultural and Food Biotechnology-State Research Institute, Rakowiecka 36, 02-532 Warsaw, Poland; renata.choinska@ibprs.pl

[2] Department of Chemistry, Biology and Biotechnology, Faculty of Civil and Environmental Sciences, Bialystok University of Technology, Wiejska 45E Street,15-351 Bialystok, Poland; e.golebiewska@pb.edu.pl

* Correspondence: paulina.walencik@ibprs.pl (P.K.W.); m.kalinowska@pb.edu.pl (M.K.)

Abstract: For many years, metal-flavonoid complexes have been widely studied as part of drug discovery programs, but in the last decade, their importance in materials science has increased significantly. A deep understanding the role of metal ions and flavonoids in constructing simple complexes and more advanced hybrid networks will facilitate the assembly of materials with tailored architecture and functionality. In this review, we highlight the most essential data on metal–flavonoid systems, presenting a promising alternative in the design of hybrid inorganic–organic materials. We focus mainly on systems containing $Cu^{II/I}$ and $Fe^{III/II}$ ions, which are necessary in natural and industrial catalysis. We discuss two kinds of interactions that typically ensure the formation of metal-flavonoid systems, namely coordination and redox reactions. Our intention is to cover the fundamentals of metal-flavonoid systems to show how this knowledge has been already transferred from small molecules to complex materials.

Keywords: hybrid materials; redox; flavonoids; transition metals

Table S1. Structural and physicochemical details on Cu^{II}/Cu^{I}-flavonoid systems established by coordination or redox interactions.

	Cu^{II}/Cu^{I}-flavonoid interaction						
**Studied system**	**Coordination**				**Redox**		
	**Coordination sites**	**Conditions**	**Physicochemical data**	**Ref.**	**Studied reaction**	**Conditions**	**Ref.**
Cu^{II}-naringenin	4-C=O/5-OH	H_2O-DMF mixture or DMF solution	UV-Vis: band II at λ_{max}= 290 nm charge transfer transitions at λ_{max}= 360 nm	[49]	$Cu^{II} + e^- \rightleftarrows Cu^I$ catalysed by naringenin	pH 4.50 and 5.50 established with acetate buffer<br>pH 6.80 and 7.50 established with HEPES or Tris-HCl buffer; gradual and rising profile of reaction specifically at higher pH	[64]
	2x naringenin *via* 4-C=O/5-OH & H_2O or DMF	Solid phase		[49]			
Cu^{II}-apigenin	4-C=O/5-OH	DMSO or MeOH solution	UV-Vis: hypsochromic shift in band II and batochromic shift in band I	[22]	$Cu^{II} + e^- \rightleftarrows Cu^I$ catalysed by apigenin	pH 4.50 and 5.50 established with acetate buffer<br>pH 6.80 and 7.50 established with HEPES or Tris-HCl buffer; gradual and rising profile of reaction specifically at higher pH	[64]
Cu^{II}-luteolin	4-C=O/5-OH & 3`-OH/4`-OH	0.16 M NaCl solution	UV-Vis: hypsochromic shift in band II (Δ ~ 15 nm)	[52]	$Cu^{II} + e^- \rightleftarrows Cu^I$ catalysed by luteolin	pH 4.50 and 5.50 established with acetate buffer<br>pH 6.80 and 7.50 established with HEPES or Tris-HCl buffer; bell-shaped response of the reaction yield regardless of pH	[64]
	pH < 5.00 no coordination<br>pH 5.00-5.80 4-C=O/5-OH<br>pH 6.00-7.20 3`-OH/4`-OH		UV-Vis at pH 5.00-5.80: hypsochromicmshift in band II and new peak at 290 nm; band I unchanged<br>UV-Vis at pH 6.00-7.20: hypsochromic shift in band II (Δ ~ 60 nm) and batochromic shift in band I (Δ ~ 4 nm)	[14]			
Cu^{II}-kaempferol	4-C=O/5-OH in DMSO<br>4-C=O/3-OH in MeOH	DMSO and MeOH solution	EPR in DMSO at 100K<br>$g_{\perp}$= 2.073<br>$g_{\parallel}$= 2.291 $A_{\parallel}$= 125 and $g_{\parallel}$= 2.242 $A_{\parallel}$= 118<br>UV-Vis in DMSO at RT: new band at 290 nm<br>UV-Vis in MeOH at RT: minor shift in band II (Δ ~ 5 nm); shift in band I (Δ~ 65 nm)	[22]	$Cu^{II} + e^- \rightleftarrows Cu^I$ catalysed by kaempferol	pH 4.50 and 5.50 established with acetate buffer<br>pH 6.80 and 7.50 established with HEPES or Tris-HCl buffer; bell-shaped response of the reaction yield regardless of pH	[64]

Cu^{II}-quercetin	mononuclear complex 4-C=O/3-OH in MeOH	MeOH solution and DMSO-d_6 for ^{1}H- NMR	UV-Vis of binuclear complex: batochromic shift in band I and II	[55]	$Cu^{II} + e^- \rightleftarrows Cu^I$ catalysed by quercetin	pH 7.50 at various M:L 1:1, 2:1, and 3:1	[66,67]
	binucelar complex 4-C=O/3-OH and 3`-OH/4`-OH in MeOH		^{1}H-NMR: pure quercetin δ 9.60 (s,^{1}H, 3-OH) and lack of this signal in binuclear complex				
					$Cu^{II} + e^- \rightleftarrows Cu^I$ catalysed by quercetin	pH 4.50 and 5.50 established with acetate buffer pH 6.80 and 7.50 established with HEPES or Tris-HCl buffer; bell-shaped response of the reaction yield regardless of pH	[64]
	mononuclear complex 3`-OH/4`-OH in H_2O	H_2O solution, pH 10.00	UV-Vis of mononuclear complex: decrease in the intensity of band I	[56]			
	binucelar complex 4-C=O/5-OH and 3`-OH/4`-OH in H_2O		UV-Vis of binuclear complex: decreasy in the intenisty of band I and band II				
	3`-OH/4`-OH at M:L below 0.0028	H_2O solution, pH 5.00	UV-Vis at M:L below 0.0028: increase in the intensity of band I	[57]			
	4-C=O/3-OH at M:L in the range from 0.0028 to 3.2		UV-Vis at M:L in the range 0.0028 – 3.2: bathochromic shift of band II				
Cu^{II}-catechin	pH 5.50: no coordination pH 7.40: 3`-OH/4`-O	H_2O solution, pH 5.50 and 7.40	UV-Vis at pH 7.40: batochromic shift and small decrease in band I (Δ ~ 16 nm)	[15]	$Cu^{II} + e^- \rightleftarrows Cu^I$ catalysed by catechin	pH 4.50 and 5.50 established with acetate buffer pH 6.80 and 7.50 established with HEPES or Tris-HCl buffer; gradual and rising profile of reaction specifically at higher pH	[64]
					$Cu^{II} + e^- \rightleftarrows Cu^I$ catalysed by catechin	pH 7.50 at M:L 2:1	[65]
Cu^I-quercetin	4-C=O/3-OH	MeOH solution	UV-Vis: batochromic shift in band I (Δ ~ 24 nm) and band II (Δ ~ 34 nm)	[70]			

Table S2. Structural and physicochemical details on Fe^{III}/Fe^{II}-flavonoid systems established by coordination or redox interactions.

Studied system	Fe^{II}/Fe^{III}-flavonoid interaction						
	Coordination				Redox		
	**Coordination sites**	**Conditions**	**Physicochemical data**	**Ref.**	**Studied reaction**	**Conditions**	**Ref.**
Fe^{III}-naringenin	4-C=O/5-OH	H_2O solution, 0.5 M $NaClO_4$ ionic strength, 25°C, pH range 1–3	Kinetic studies based on UV-Vis	[95]	$Fe^{II} - e^- \rightleftarrows Fe^{III}$ catalysed by naringenin	H_2O solution at pH 6.50 and 7.00	[59]
	4-C=O/5-OH	H_2O solution, pH 6.50	UV-Vis: bathochromic shift in band I (λ_{max}= 350 nm, sh)	[60]		DMSO solution	[69]
Fe^{III}-apigenin	4-C=O/5-OH	H_2O solution, pH 2.00	UV-Vis: Ligand-to-Fe^{III}-Charge Transfer (LMCT) transitions (λ_{max}= 520 nm); bathochromic shift in band I and II	[59]			
Fe^{III}-luteolin	3`-OH/4`-OH	EtOH solution	UV-Vis: bathochromic shift in band I and II	[61]	$Fe^{III} + e^- \rightleftarrows Fe^{II}$ catalysed by luteolin	H_2O solution, 90°C	[70]
	3`-OH/4`-OH	H_2O solution, pH 6.50	UV-Vis: bathochromic shift in band I (λ_{max}= 420 nm) and II (λ_{max}= 270 nm) and new band from $\pi \rightarrow d_\pi$ LMCT (λ_{max}= 550 nm)	[60]			
Fe^{III}-kaempferol	4-C=O/5-OH	H_2O solution, pH 4.00 and 8.00	UV-Vis: bathochromic shift in band I	[62]			
	4-C=O/5-OH	H_2O solution, pH 6.50	UV-Vis: bathochromic shift in band I (λ_{max}= 420 nm)	[60]			
Fe^{II}-quercetin	4-C=O/5-OH	MeOH solution	UV-Vis: bathochromic shift in band I and band II; new band from $\pi \rightarrow d_\pi$ LMCT (λ_{max}= 425 nm)	[78]			
Fe^{III}-quercetin	3`-OH/4`-OH in the dominant species $Fe(H)_{-5}(H_5Que)_2^{2-}$	H_2O solution, pH range 2.00-4.50, 0.16 M NaCl, 37°C $Fe(H)_{-5}(H_5Que)_2^{2-}$ species with M:L 1:2, pH 2.00 $Fe(H)_{-4}(H_5Que)^-$ with M:L 1:1, pH 3.00 $Fe(H)_{-6}(H_5Que)_2^{3-}$ with M:L 1:2, pH 3.00	Potentiometric titration and DFT	[80]	$Fe^{III} + e^- \rightleftarrows Fe^{II}$ catalysed by quercetin	MeOH-H_2O mixture 1:1 with 0.1 % acetic acid; final pH 3.00	[71]
	4-C=O/3-OH	DMSO-d_6	^{1}H-NMR, ^{13}C-NMR and maps of the molecular electrostatic potential (MEP)	[80]	$Fe^{III} + e^- \rightleftarrows Fe^{II}$ catalysed by quercetin	0.1 M HCl-MeOH 1:1, 37°C	[72]
					$Fe^{III} + e^- \rightleftarrows Fe^{II}$ catalysed by quercetin	MeOH-H_2O mixture 1:1 under aerobic and anaerobic atmosphere	[74]

					$Fe^{III} + e^{-} \rightleftarrows Fe^{II}$ catalysed by quercetin	50 mM acetate buffer, pH 5.50	[15]
Fe^{III}-catechin	3\`-OH/4\`-OH	aqueous solution, pH 7.40	UV-Vis; new LMCT band at λ_{max}= 491 nm, pH range 9.71-10.10 and λ_{max}= 551 nm, pH 7.40	[82]	$Fe^{III} + e^{-} \rightleftarrows Fe^{II}$ catalysed by catechin	nano-ESI–MS analysis; MeOH- H_2O mixture 1:1	[54]
